# 6.3 EEG AS A TOOL FOR PSYCHOSIS RESEARCH: CHALLENGES, PITFALLS AND NEW OPPORTUNITIES

**DOI:** 10.1093/schbul/sby014.020

**Published:** 2018-04-01

**Authors:** Christoph Mulert

**Affiliations:** University Hospital Hamburg Eppendorf

## Abstract

**Background:**

Electroencephalography (EEG) as the oldest technique currently in use for the analysis of brain function has strong advantages not offered by other techniques: it is a direct measurement of neuronal activity and offers a high temporal resolution. Accordingly, it is very useful for the investigation of neuronal oscillations which are related to disturbed core mechanisms of schizophrenia such as NMDA-receptor dysfunction or E/I imbalance and alterations in connectivity.

**Methods:**

On the other hand, the method has also strong limitations, e.g. the difficulty of precise localization, which is due to the inverse problem and also its blindness to subcortical structures that are highly relevant for psychosis research, such as the ventral striatum.

**Results:**

Uncritical use of this technique has created widespread skepticism, leading probably to some degree of underestimation of the unique opportunities offered. In this talk, limitations of the technique will be addressed as well as current strategies of proper usage such as the combination of EEG and fMRI.

**Discussion:**

Simultaneous EEG-fMRI offers the best from both modalities, that is high temporal and high spatial resolution, but here, too, methodological challenges have to be addressed. Finally, the development of new noninvasive tools for brain stimulation such as transcranial alternating current stimulation (tACS) with the opportunity of frequency-specific modulation of neuronal oscillations (“entrainment”) for both brain research and therapy makes detailed information about disturbed oscillations patterns in psychosis even more relevant.

